# Functional analysis of ESM1 by shRNA-mediated knockdown of its expression in papillary thyroid cancer cells

**DOI:** 10.1371/journal.pone.0298631

**Published:** 2024-04-16

**Authors:** Lijun Xie, Limeng He, Wei Zhang, Hao Wang

**Affiliations:** 1 Department of Nuclear Medicine, The First Affiliated Hospital of Kunming Medical University, Kunming, P.R. China; 2 Department of Nuclear Medicine, Sichuan Provincial People’s Hospital, University of Electronic Science and Technology of China, Chengdu, P.R. China; Tehran University of Medical Sciences, ISLAMIC REPUBLIC OF IRAN

## Abstract

**Objective:**

Endothelial specific molecule-1 (ESM1) is implicated as an oncogene in multiple human cancers. However, the function of ESM1 in papillary thyroid cancer (PTC) is not well understood. The current study aimed to investigate the effect of ESM1 on the growth, migration, and invasion of PTC to provide a novel perspective for PTC treatment.

**Methods:**

The expression levels of ESM1 in PTC tissues form 53 tumor tissue samples and 59 matching adjacent normal tissue samples were detected by immunohistochemical analysis. Knockdown of ESM1 expression in TPC-1 and SW579 cell lines was established to investigate its role in PTC. Moreover, cell proliferation, apoptosis, wound healing, and transwell assays were conducted in vitro to assess cell proliferation, migration and invasion.

**Results:**

The findings revealed that ESM1 expression was significantly higher in PTC tissues than that found in paraneoplastic tissues (P<0.0001). Knockdown of ESM1 expression inhibited the proliferation, migration, and invasion of TPC-1 and SW579 cells in vitro. Compared with the control group, the mRNA and protein levels of ESM1 in PTC cells were significantly reduced following knockdown of its expression (P<0.01). In addition, ESM1-knockdown cells indicated decreased proliferation and decreased migratory and invasive activities (P<0.01, P<0.01, P<0.001, respectively).

**Conclusions:**

ESM1 was identified as a major gene in the occurrence and progression of PTC, which could increase the proliferation, migration, and invasion of PTC cells. It may be a promising diagnostic and therapeutic target gene.

## Introduction

Thyroid cancer, with an estimated 586,202 new cases worldwide in 2020 alone, stands as a significant public health concern [1]. Its incidence, particularly in affluent regions, has experienced a marked increase, attributed to improved detection methods and environmental factors [2]. The disease is multifactorial, with a complex interplay between genetic predispositions and various environmental factors, such as exposure to carcinogens, dietary patterns, and lifestyle choices [3]. Despite the prominence of surgery, supplemented by radiotherapy or drug therapy, as the primary treatment modalities, these approaches do not guarantee freedom from recurrence or metastasis, which are common postoperative complications that significantly compromise quality of life [4]. Therefore, there is a need to develop supportive alternative diagnostic and therapeutic approaches.

Endocan, known formally as Endothelial Cell-Specific Molecule-1 (ESM1), is a dermatan sulfate proteoglycan first identified in a cDNA library from human endothelial cells in 1996. This soluble 50 kDa proteoglycan, constituted by 165 amino acids and a single dermatan sulfate chain, can be detected circulating in the bloodstream [5]. It is noteworthy for its role in the pathogenesis of various cancers, where its overexpression is closely tied to tumor growth, progression, and angiogenesis. In colorectal, gastric, and hepatocellular carcinomas, ESM1 has been found to significantly influence cell proliferation and migration, indicating its involvement in the aggressive behavior of these malignancies [6–8]. However, the literature on ESM1’s impact on thyroid cancer remains sparse, leaving its potential as a prognostic marker and therapeutic target largely untapped.

The current study seeks to fill this void in the literature by utilizing shRNA to knock down ESM1 in thyroid cancer cell lines and conducting a comprehensive analysis of its role. We measured ESM1 gene expression and evaluated its association with cell proliferation, apoptosis, migration, and invasion. Our aim is to provide a nuanced understanding of ESM1’s involvement in thyroid cancer, which may facilitate its use as a significant biomarker for the disease. The outcomes of these experiments are anticipated to constitute a foundational step towards characterizing ESM1’s role in the etiology and progression of papillary thyroid carcinoma, with broader implications for the development of innovative diagnostic and therapeutic strategies.

## Materials and methods

### Source of specimens

From December 2020 to June 2021, 53 tumor tissue samples (confirmed by pathological diagnosis) and 59 matching adjacent normal tissue samples (at least 5 cm away from the tumor edge) were collected from papillary thyroid carcinoma tissues extracted from patients who underwent surgical resection in thyroid surgery in the Sichuan Provincial People’s Hospital. The present study was subject to approval (approval no. 2020‑203) by Sichuan Provincial People’s Hospital Ethics Committee and all patients provided informed consent. The patients younger than 18 years old who received preoperative radiotherapy, chemotherapy, or biological therapy were excluded. Postoperative TNM staging in all cases was made in accordance with the international TNM staging standard (8th edition) formulated by the International Union Against Cancer and the American Joint Committee on Cancer (UICC/AJCC) [9].

### Immunohistochemical (IHC) analysis

Hematoxylin and eosin (H&E) staining was conducted on papillary thyroid carcinoma and matched adjacent normal tissues for tumor diagnosis and the expression of ESM1 was detected by IHC analysis. The tissue sections were incubated with anti-ESM1 rabbit polygonal antibody (cat. no. ab103590, Abcam) overnight at 4˚C. The following day, the samples were incubated with a secondary antibody (cat. no. A0208, Beyotime Institute of Biotechnology) at room temperature for 60 min. ESM1 detection was performed by IHC. All slides were independently evaluated by two experienced pathologists. The IHC stain was described as a brown color in the tumor cell cytoplasm. ESM1 expression levels were assessed by the immunoreactivity score, which is equivalent to the percentage of positive cells multiplied by the staining intensity [10].

### Cell culture

Human PTC cell lines (TPC-1, SW579 and BCPAP) and human thyroid follicular epithelial cell line (Nthy-ori3-1) were obtained from YBR Bioscires (Shanghai, China). Dulbecco’s modified Eagle’s medium (DMEM, Corning) mixed with 10% fetal bovine serum (FBS, Ausbian), 100 U/mL penicillin (Gibco), and 100 μg/mL streptomycin (Gibco) was utilized for cell culture. Cells were incubated at 37°C with 5% CO_2_ in a humidified atmosphere.

### Knockdown of ESM1 expression in thyroid cancer cells

shRNA interference technology was used in this experiment. The specific interference sequence used targeting ESM1-1 was the following: 5′-ATGAGGAAATGGTTAAATCCA-3; the exclusive interference sequence of ESM1-2 was as follows: 5′-TGGCAATATTGTGAGAGAAGA-3; the exclusive interference sequence of ESM1-3 was as follows: 5′-GGCCGCCTGGAGCAATAATTA-3. The aforementioned ESM1 target fragments were inserted into the Age Ⅰ and EcoR Ⅰ sites of the BR-V108 vector to construct the lentivirus (LV)-ESM1-green fluorescent protein (GFP)-shRNA interference group (LV-shESM1) and the LV-Ctrl-GFP-shRNA negative control group (LV-shCtrl). These reagents were purchased from Shanghai Yiberui Biomedical Technology Co., Ltd.; the positive clones were screened and identified.

Prior to the infection, TPC-1 and SW579 cells in the logarithmic growth phase were incubated in 6-well plates at a density of 2x10^5^ cells and cultured in a cell incubator. The cells that were 60% confluent were used for the interference experiments. The cells were split into the negative control and experimental groups and were infected with LV-shESM1 and LV-shCtrl respectively. A total of 8 and 4 μl of retroviral sample was incubated with a concentration of 3x10^8^ TU/ml and used to infect TPC-1 and SW579 cells. Following 72 h of incubation, the expression of GFP in TPC-1 and SW579 cells was observed using a fluorescence microscope, and the infection efficiency was calculated.

### Total RNA isolation and cDNA synthesis

The cells were seeded at a density of 1x10^5^ cells in 12-well plates in 1 ml culture medium without antibiotics. shRNA was transfected into the cells, which were incubated for 48 h. The cells were washed twice with 1x phosphate-buffered saline (PBS) and total RNA was separated with Trizol (Sigma-Aldrich; Merck KGaA) depending on the manufacturer’s instructions. A total of 1 μg RNA was reverse transcribed using oligo (dT) primers and the Transcript High-Fidelity cDNA Synthesis Kit (Vazyme Biotech Co., Ltd.). The following conditions were used: Incubation at 29˚C for 10 min, 48˚C for 60 min, and 85˚C for 5 min according to the manufacturer’s instructions.

### Reverse transcription-quantitative PCR (RT-qPCR)

RT-qPCR analysis was performed using the Bio-Rad CFX Connect fluorescent quantitative PCR instrument (Bio-Rad Laboratories, Inc.). The PCR mix consisted of 2x QuantiTect SYBR Green PCR Master Mix (Vazyme Biotech Co., Ltd.) containing DNA polymerase, SYBR Green I dye, dNTP mix including dUTP, 5 mM MgCl_2_, PCR buffer, 20 pmol forward and reverse primers and RNase. Free water and cDNA were mixed in the samples in a total volume of 25 μl. The housekeeping gene GAPDH was used for normalization. The following PCR conditions were used: Initial denaturation at 95˚C for 5 min followed by 42 cycles of amplification; each cycle consisted of a denaturation step at 95˚C for 30 sec, an annealing step at 58˚C for 30 sec, and a polymerization step at 72˚C for 1 min. The final stage included polymerization at 72˚C for 5 min. The following sequences were used for amplification: GAPDH FP, 5’-TGACTTCAACAGCGACACCCA-3’, RP, 5’-CACCCTGTTGCTGTAGCCAAA-3’ (121 bp); ESM1 FP 5’- TGACAGCAGTGAGTGCAAAAGC-3’, RP 5’-TGCCATCCATGCCTGAGACT-3’ (128 bp). To verify the size of the amplified fragments, the PCR products were separated by 2.0% agarose gel electrophoresis and visualized with ethylene bromide.

### Protein separation and western blot analysis

The cells were seeded at a density of 3x10^5^ cells in 6-well plates containing 2 ml of medium without antibiotics. The shRNA sequences were transfected following 48 h of culture; the cells were washed twice with ice-cold PBS and removed from the plate. The cells were enclosed in 250 μl protease inhibitor cocktail. The protein concentration of the cell extracts was established by the Bradford assay (HyClone; Cytiva/Pierce; Thermo Fisher Scientific, Inc.). A total of 20 μg total protein was used for western blot analysis. A total of 30 μl of each sample was mixed with 10 μl 4x SDS sample buffer and SDS-PAGE was performed using a 10% polyacrylamide gel. Electrophoresis was performed at 100 and 240 V for 5 and 17 min, respectively utilizing a protein electrophoresis instrument (Shanghai Tianneng). Following electrophoresis, the proteins were transferred to a polyvinylidene fluoride membrane (MilliporeSigma) using a protein transfer apparatus (Shanghai Tianneng) in Tris-buffered saline containing 5% skimmed milk powder and containing 0.05% Tween 20 (TBS-T) for 30 min. The membranes were hybridized with an anti-ESM1 antibody (cat. no. ab103590, Abcam) [11] or an anti-GAPDH antibody (cat. no. 60004–1, Proteintech) [12] overnight at 4˚C. Following washing three times with TBS-T for 5 min at room temperature, the membranes were incubated with anti-mouse and anti-rabbit IgG, horseradish peroxidase-conjugated secondary antibody (cat. no. A0216; A0208; Beyotime Institute of Biotechnology) for 40 min at room temperature. The membranes were subsequently washed three times with TBS-T for 5 min at room temperature and at last developed by using the enhanced chemiluminescence system (ECL; Bio-Rad Laboratories, Inc.).

### Cell growth activity assay

To determine cell viability, the cells were prepared and transformed as described above. The absorbance values were measured following transfection to assess the effect of ESM1 knockdown on cell viability. TPC-1 and SW579 cells were uniformly incubated in 96-well plates at 5x10^3^ cells, and each well was developed with 5 replicate wells. After culturing for 1, 2, 3, 4, and 5 days respectively, MTT was added to each well at a final concentration of 5 mg/ml, and the cells were further incubated at 37˚C for 4 h. Dimethyl sulfoxide (100 μl) was added to each well after removing the medium. After shaking the plates for 5 min, the absorbance of the mixture was measured at 570 nm with a spectrophotometer.

### Cell proliferation and apoptosis assay

The aforementioned cells were incubated in 6-well plates. The cells were collected following 48 h of culture, washed twice with pre-cooled D-Hanks solution, and fixed with 70% ethanol overnight at 4˚C. Each group was centrifuged at 1,000xg for 5 min, washed with D-Hanks solution, stained with 1 ml PI staining solution (Sigma-Aldrich; Merck KGaA), and incubated at room temperature in the dark for 30 min. Cell proliferation was analyzed by flow cytometry (MilliporeSigma, Guaveasy Cyte HT).

The apoptotic rate was evaluated using the Annexin V-FITC/propidium iodide (PI) Apoptosis Detection kit (Sigma-Aldrich; Merck KGaA) according to the instructions provided by the manufacturer. The cells were seeded into 6-well culture plates (4x10^5^ cells). Following treatment, the cells were collected, washed with PBS, and resuspended in 500 μl binding buffer. Subsequently, 5 μl Annexin V-FITC and 5 μl PI were added to the buffer and incubated at room temperature for 15 min in the dark. The cells were analyzed by flow cytometry (MilliporeSigma).

### Transwell assay

The cells (1x10^5^) were seeded into 6-well plates. When the cell density reached 100%, the wells were scraped vertically along the central axis with a 1 ml pipette tip to wash away the detached cells. The 6 or 24 h wound healing status was imaged under a light microscope and the percent wound area was calculated.

### Migration assay

TPC-1 and SW579 cells (2x10^5^ cells/ml) were seeded into transwell plates. A total of 200 μl cell suspension was added to each chamber; the upper and lower transwell chambers were incubated for 24 h with serum-free medium and 600 μl medium containing 10% FBS, respectively. The cells were fixed with methanol and stained with crystal violet. At last, the assay was completed by measuring the number of migrated cells.

### Invasion assay

A protocol similar to the migration assay was used, with the exception that the transwell units were pre-coated with 200 μg/ml Matrigel (Sigma-Aldrich; Merck KGaA) and incubated overnight.

### Statistical analysis

The experimental data were analyzed using SPSS 21.0 software. All results were supported by at least three independent experiments. The measurement data were expressed as mean ± standard deviation (x ± s) and the distribution abnormalities or variance heterogeneity were analyzed by Mann Whitney test. P<0.05 was considered to indicate a statistically significant difference.

## Results

### ESM1 gene expression in cancerous and paraneoplastic tissues of the thyroid

**Fig 1:**
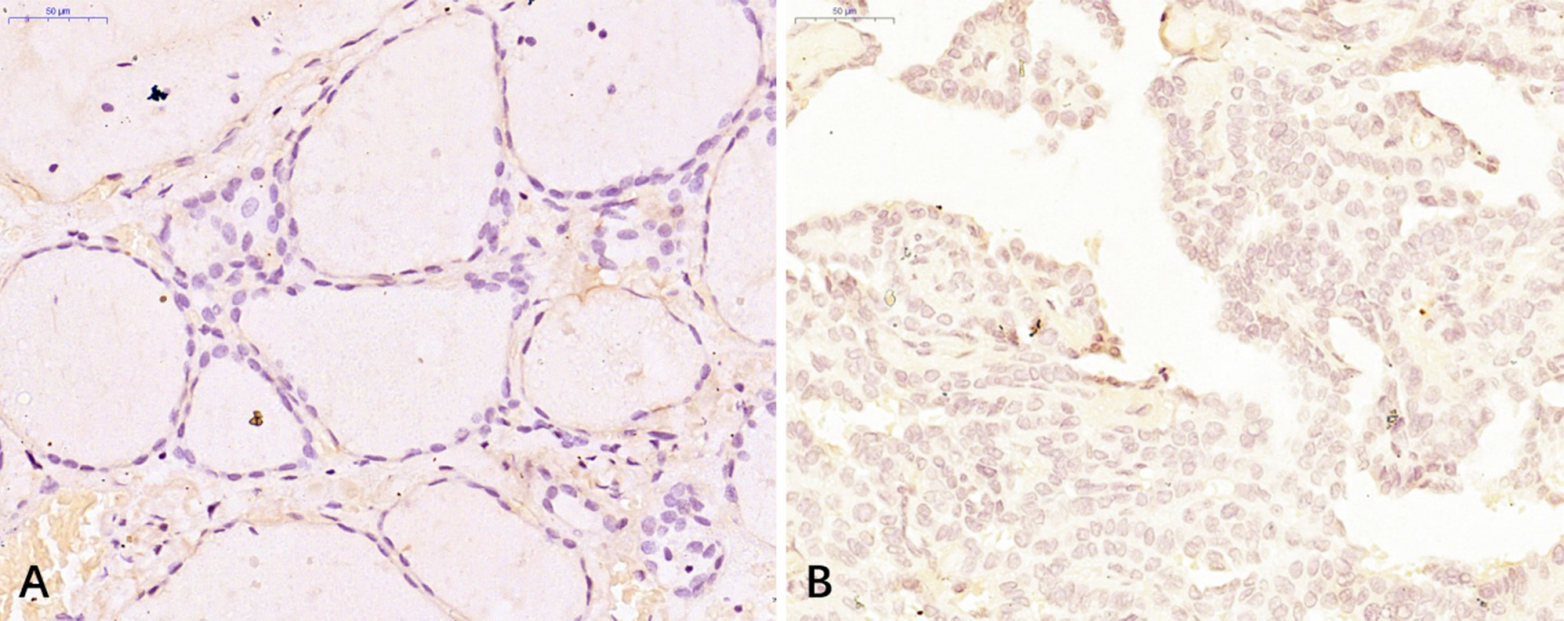
Immunohistochemistry of PTC tissues and paraneoplastic tissues (observed under light microscope: ×400). A: paraneoplastic tissue; B: thyroid cancer tissue.

Among 53 patients with PTC, the expression levels of the EMS1 gene in thyroid cancer tissues were markedly higher than those noted in adjacent tissues (P<0.0001; Table 1 and Fig 1).

**Table 1:** Expression patterns in PTC tissues and para-carcinoma tissues revealed in immunohistochemistry analysis.

EMS1 expression	Tumor tissue		Para-carcinoma tissue		p value
	Cases	Percentage	Cases	Percentage	0.000***
Low	22	41.5%	59	100.0%	
High	31	58.5%	0	-	

### Determination of ESM1 expression in thyroid cancer cells and identification of effective interference targets

**Fig 2:**
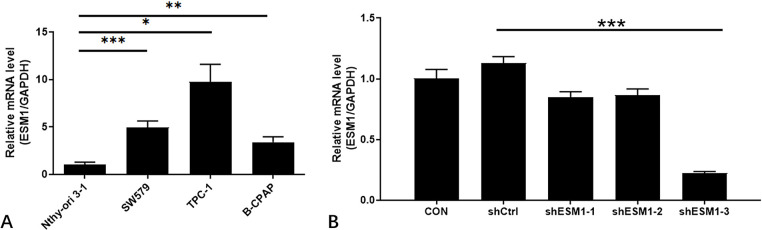
EMS1 expression in PTC and the results of effective interference target screening. A: The expression of ESM1 gene was higher in SW579, TPC-1 and B-CPAP cells than in Nthy-ori 3–1 cells. B: The endogenous screening experiments on shESM1 sequences designed with three different targets in TPC-1 cells showed that shESM1-3 had a higher knockdown efficiency on ESM1 than the control group. *P < 0.05, **P< 0.01, *** P <0.001.

RT-qPCR analysis was used to detect the baseline expression of ESM1 in Nthy-ori 3–1, SW579, TPC-1, and BCPAP cells (Fig 2A). The expression levels of the ESM1 gene in SW579, TPC-1, and BCPAP cells were relatively high (P<0.05). The levels were notably high in TPC-1 cells. Therefore, the TPC-1 cell line was utilized to carry out endogenous screening experiments based on three different designed shESM1 sequences (Fig 2B). The results of the RT-qPCR analysis indicated that the knockdown efficiency of shESM1-3 on ESM1 was higher and reached 80.4% (P<0.001). Therefore, the retroviral vector expressing shESM1-3 was selected as a gene knockdown tool in subsequent experiments.

### Knockdown efficiency of thyroid cancer cells

**Fig 3:**
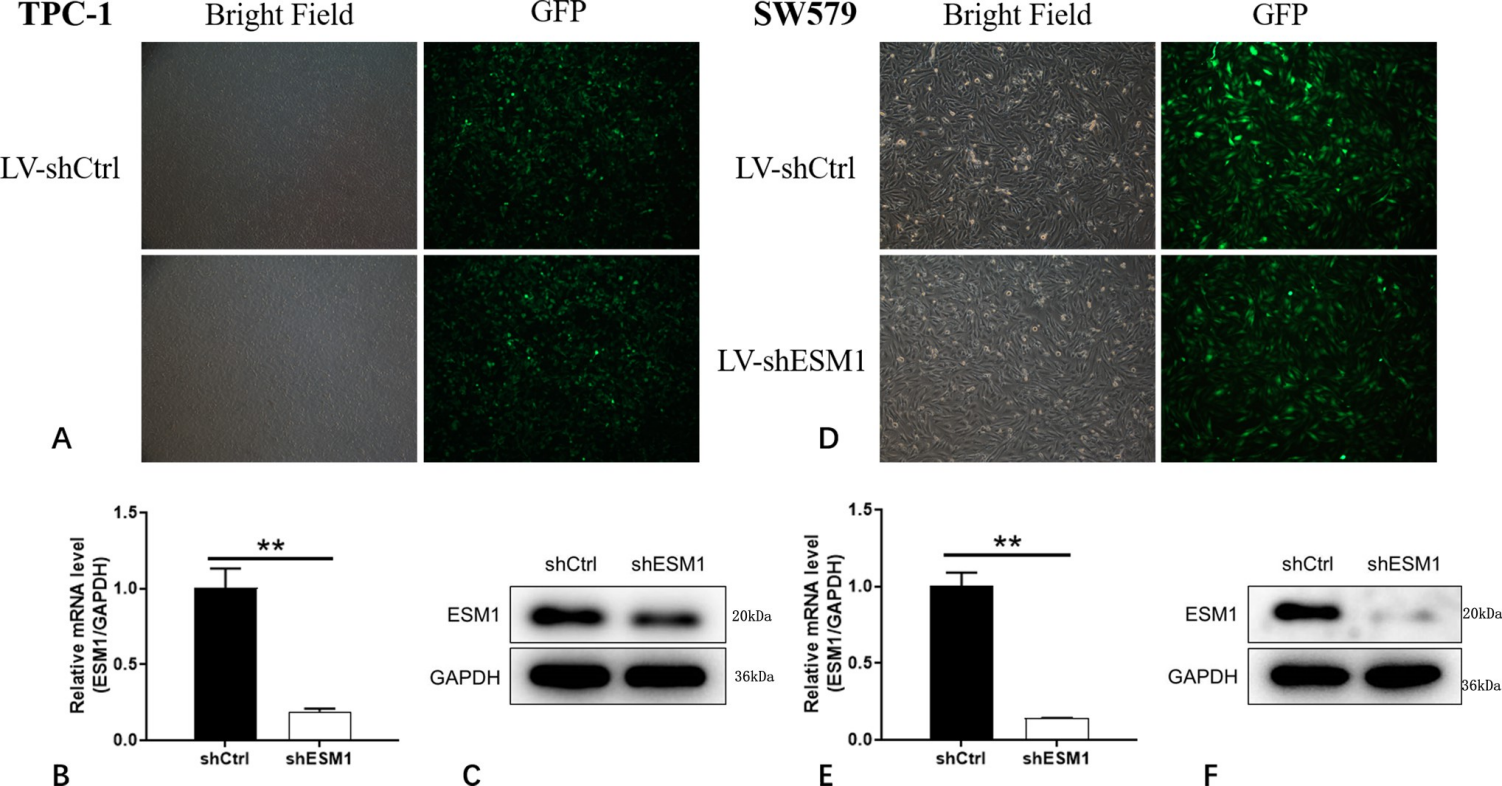
Results of knockdown efficiency of ESM1 in TPC-1 and SW579 cell lines. A: The efficiency of TPC-1 cell infection by detecting the fluorescent signal of the GFP tag carried by the ESM1 knockout retroviral vector shESM1. B: After lentivirus infection, ESM1 expression in TPC-1 cells was significantly reduced in the shESM1 group compared with the shCtrl group. C: The results of Western blot showed that the ESM1 protein level of TPC-1 cells in the shESM1 group was down-regulated compared with the shCtrl group after retrovirus infection. D: The efficiency of SW579 cell infection by detecting the fluorescent signal of the GFP tag carried by the ESM1 knockout retroviral vector shESM1. E: After lentivirus infection, ESM1 expression in SW579 cells was significantly reduced in the shESM1 group compared with the shCtrl group. F: The results of Western blot showed that the ESM1 protein level of SW579 cells in the shESM1 group was down-regulated compared with the shCtrl group after retrovirus infection. **P< 0.01. S1 Raw images.

Following selection of the TPC-1 and SW579 cells as the main cell lines of investigation, the retroviral vector shESM1-3 and the blank vector sh control (Ctrl) were designed and constructed. ShESM1 was used to knockdown ESM1 expression and shCtrl was used as the corresponding negative control. The imaging method was used to detect the cell infection efficiency, and the detection of the fluorescent signal of the GFP tag on the retroviral vector indicated that the transfection efficiency of all groups exceeded 80% (Fig 3A and 3D).

The knockdown efficiency of TPC-1 and SW579 cells was verified by RT-qPCR and western blot analyses. The results of the RT-qPCR analysis indicated that following retroviral infection, the knockdown efficiency of ESM1 in the shESM1 group in TPC-1 cells was 81.07% compared with that of the shCtrl group (P<0.01; Fig 3B). Among them, the knockdown efficiency of ESM1 in the shESM1 group of SW579 cells was 85.88% (P<0.01; Fig 3E). The results of the western blot analysis indicated that the ESM1 protein levels in the shESM1 group in TPC-1 and SW579 cells were downregulated compared with those of the shCtrl group following retroviral infection. (Fig 3C and 3F).

### Proliferation assay

**Fig 4:**
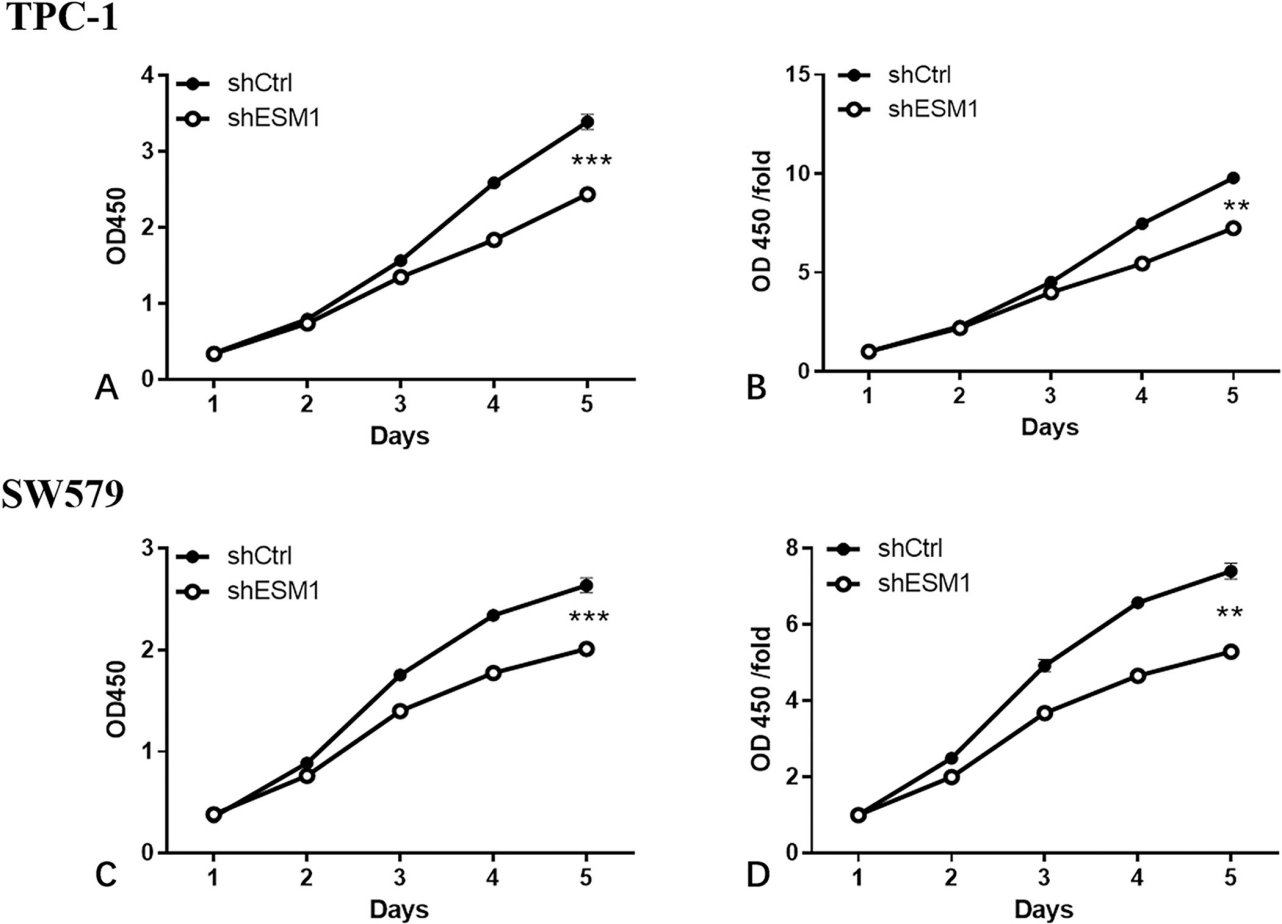
The results of cell growth. A: Comparison of the absorption rate of TPC-1 cells at wavelength 570 nm with time. B: Comparison of the fold change in absorption rate of TPC-1 cells at wavelength 570nm with time. C: Comparison of the fold change in absorption rate of SW579 cells at wavelength 570 nm with time. D: Comparison of the fold change in absorption rate of SW579 cells at wavelength 570 nm with time.

The MTT assay was utilized to detect the effects of knockdown of ESM1 expression on the proliferative ability of thyroid cancer cells. The absorbance of the control group continued to rise from day 1 to day 5, while the absorbance of the shESM1 group in TPC-1 and SW579 cells was increased from day 1 to day 3 and subsequently it was decreased from day 3 to day 5. Moreover, the number of thyroid cancer cells in the shESM1 groups was significantly lower than that in the control group from day 4 to day 5 (P<0.01; Fig 4). The data indicated that the cell proliferative abilities of TPC-1 and SW579 cells were markedly inhibited following knockdown of ESM1 expression.

### Apoptosis and cell cycle distribution assay

**Fig 5:**
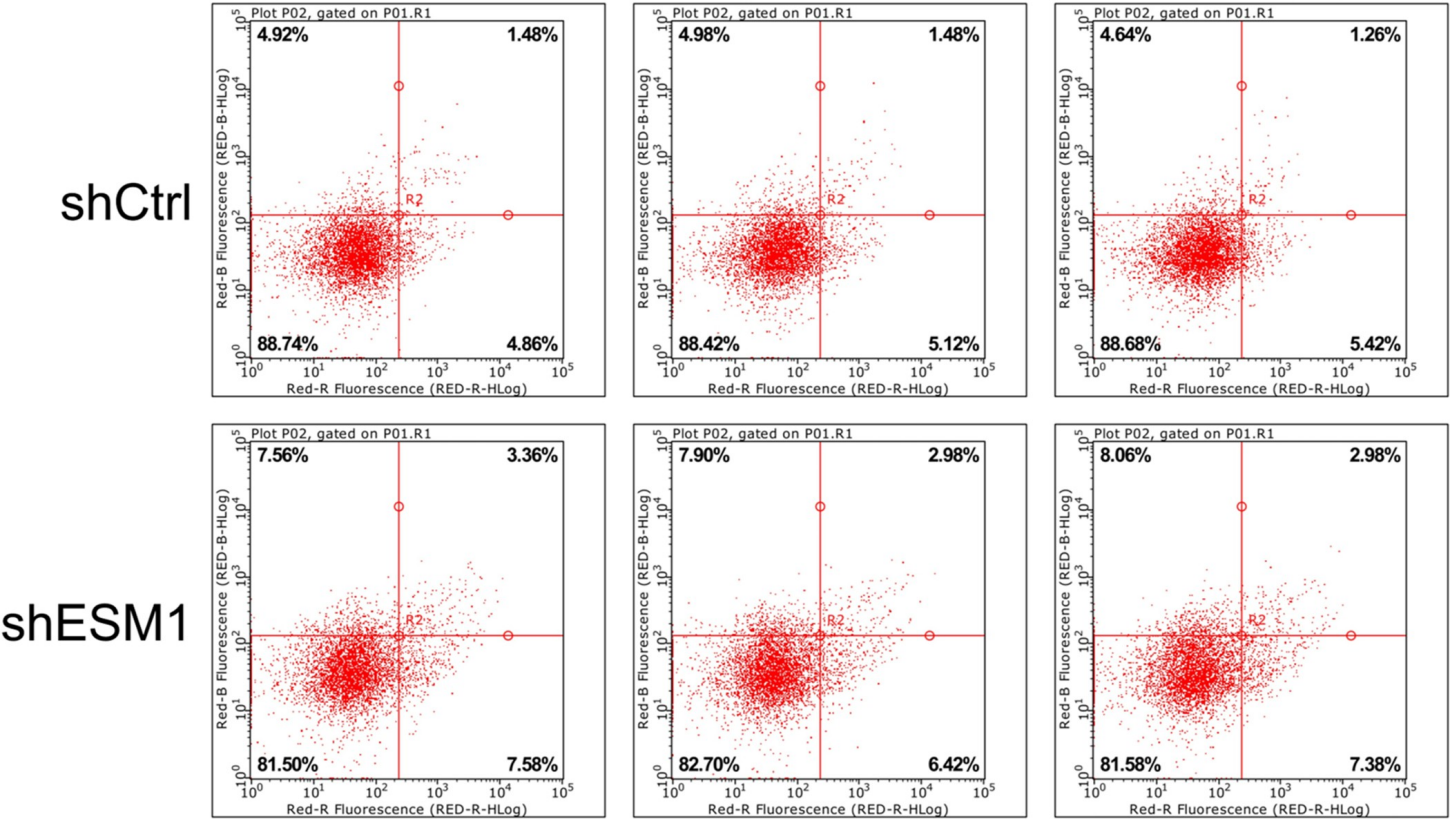
Two-dimensional graph of flow cytometry showed that ESM1 knockdown significantly promoted apoptosis in TPC-1 cells.

**Fig 6:**
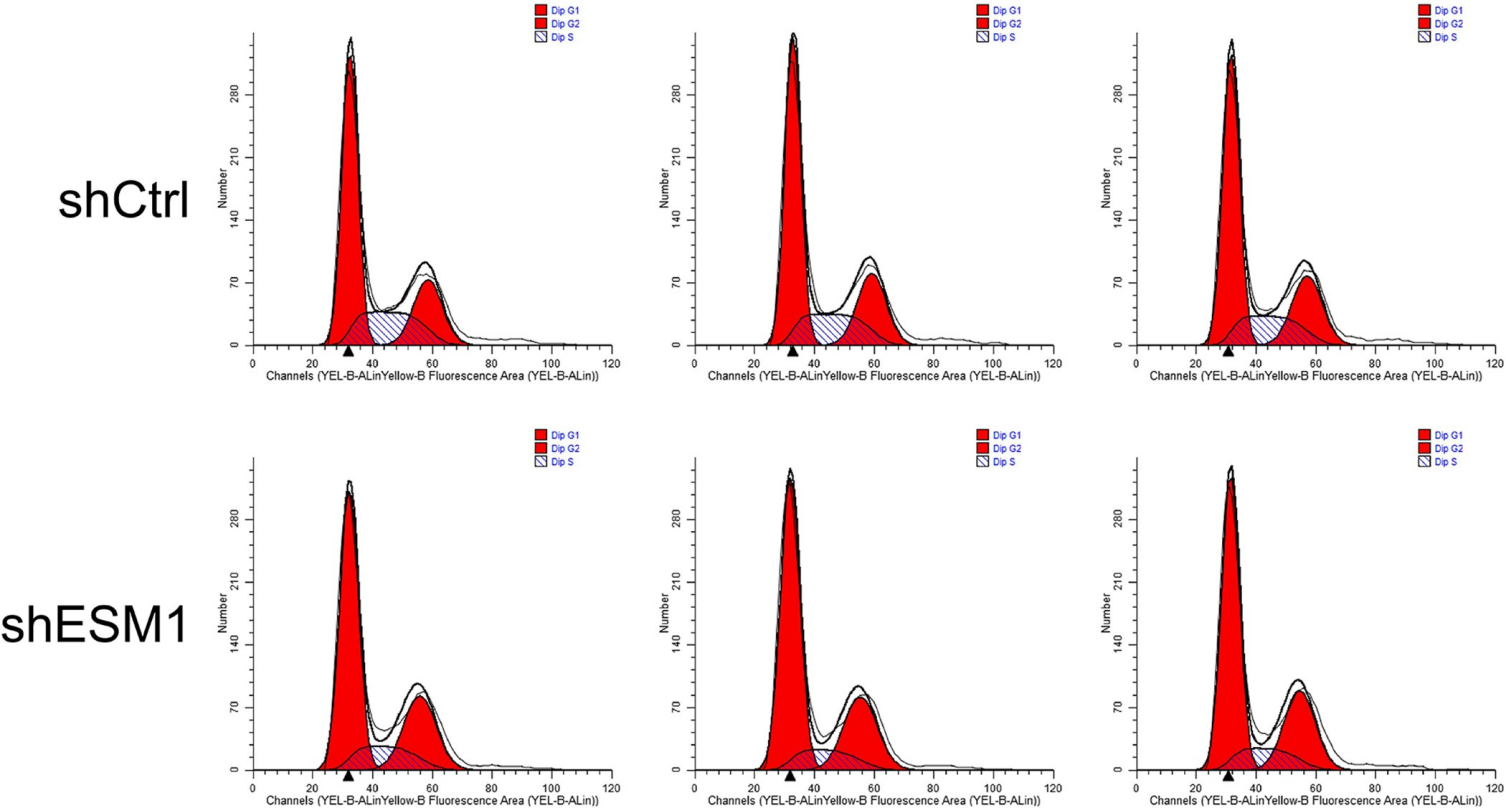
G2 phase arrest was observed in TPC-1 cells after ESM1 knockdown by flow cytometry.

**Fig 7:**
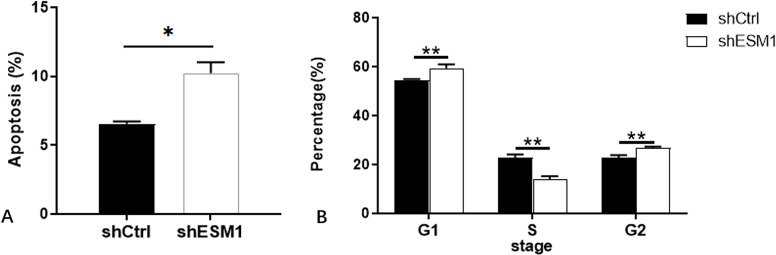
Apoptosis and cell cycle results of TPC-1 cell lines. A: The apoptosis rate of the shESM1 group in TPC-1 cells was markedly higher than that of the control group. B: Compared with shCtrl group, TPC-1 cells of shESM1 group decreased in S phase and increased in G2 phase. *P < 0.05, **P< 0.001.

**Fig 8:**
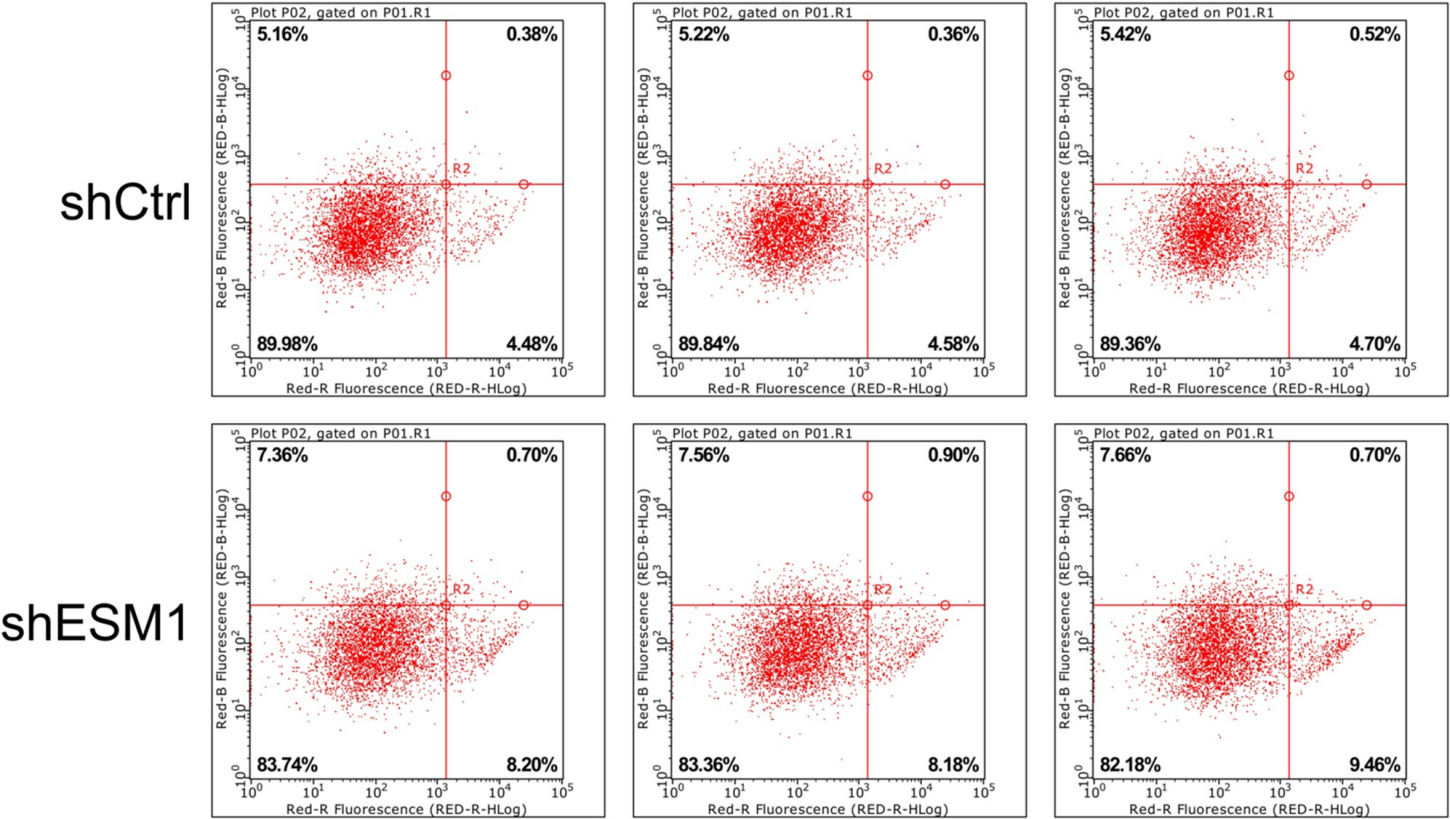
Two-dimensional graph of flow cytometry showed that ESM1 knockdown significantly promoted apoptosis in SW579 cells.

**Fig 9:**
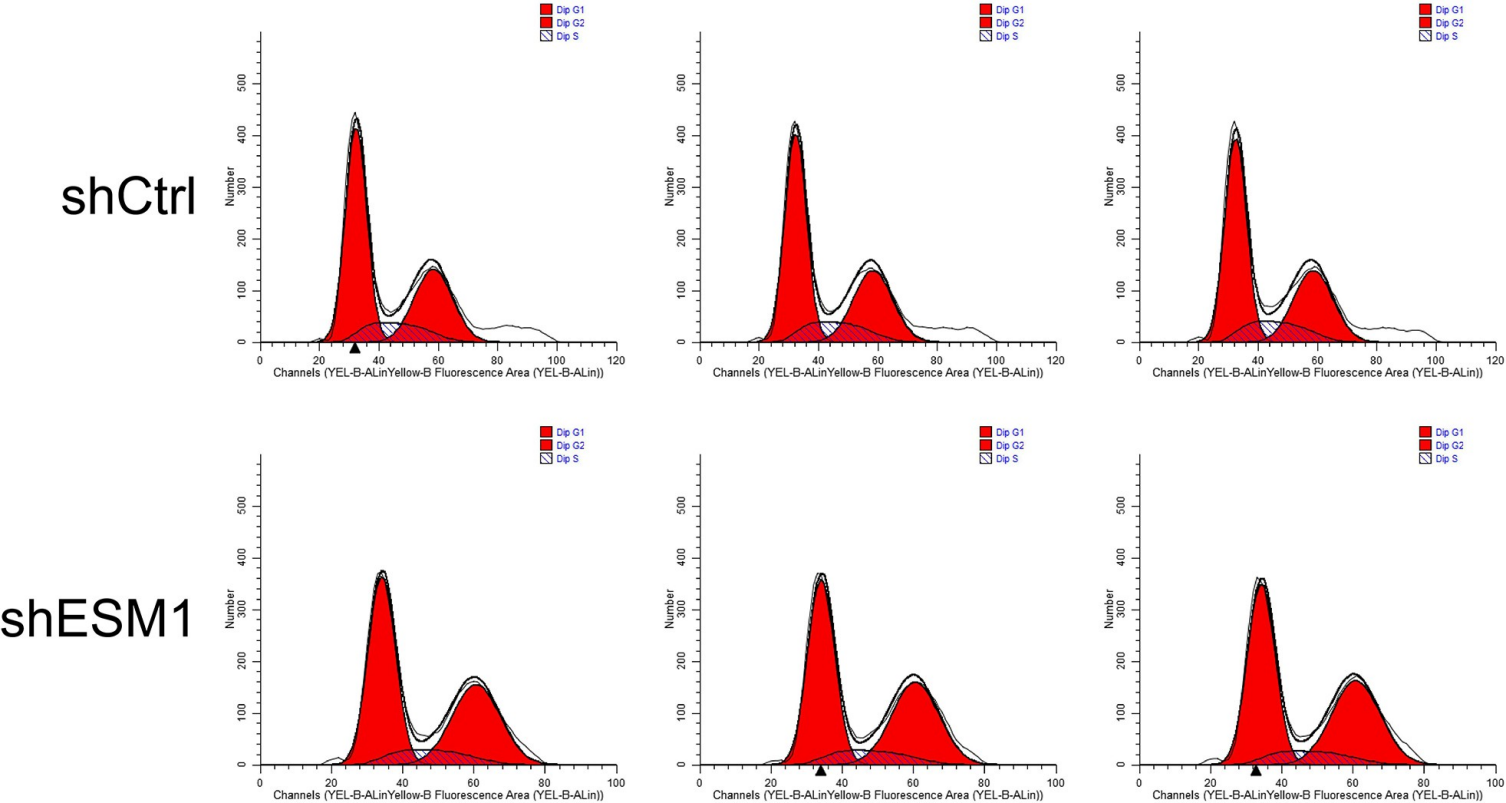
G2 phase arrest was observed in SW579 cells after ESM1 knockdown by flow cytometry.

**Fig 10:**
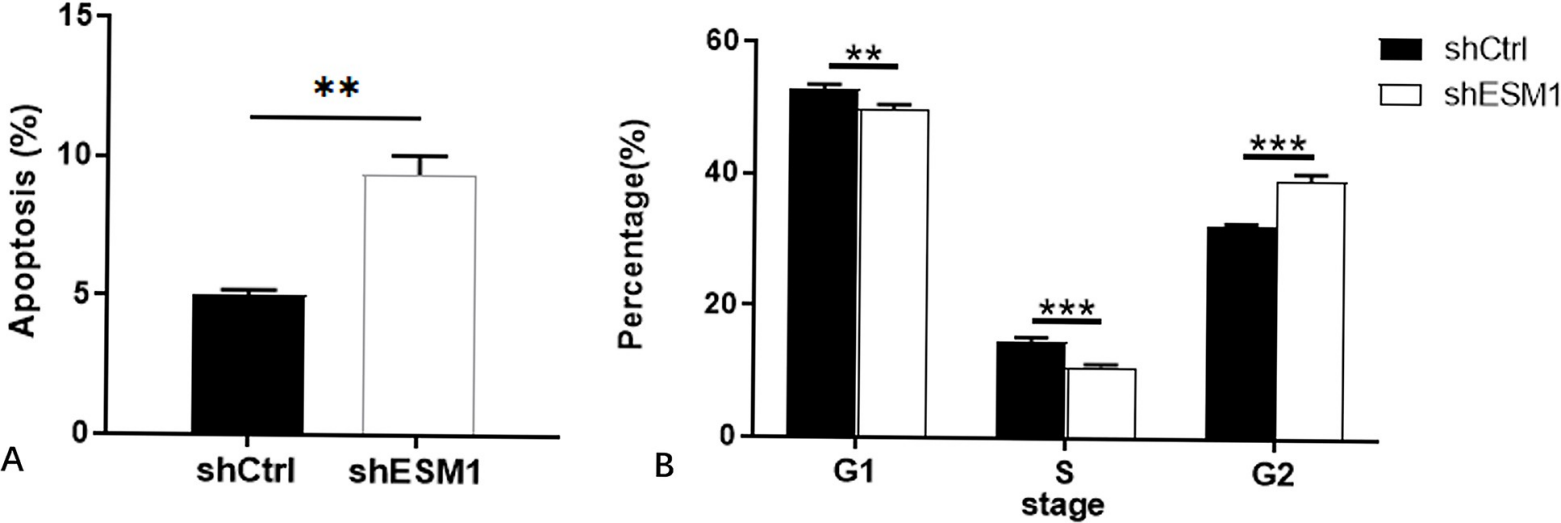
Apoptosis and cell cycle results of SW579 cell lines. A: The apoptosis rate of the shESM1 group in SW579 cells was markedly higher than that of the control group. B: Compared with shCtrl group, SW579 cells of shESM1 group decreased in S phase and increased in G2 phase. **P< 0.01, *** P <0.001.

Flow cytometry was used to identify the effects of knockdown of ESM1 expression on apoptosis and the cell cycle distribution of thyroid cancer cells. The apoptotic rates of TPC-1 and SW579 cells were examined following their transfection with shESM1 and the data indicated that they were markedly higher than those of the control group (P<0.01; Figs 5–10). Compared with the shCtrl group, the number of the cells in the S phase was decreased in the shESM1 groups of TPC-1 and SW579 cells, (P<0.01 and P<0.001, respectively). However, the number of cells in the G2 phase was increased (P<0.01 and P<0.001, respectively; (Figs 5–10). The data indicated that knockdown of ESM1 expression in TPC-1 and SW579 cells could significantly promote cell apoptosis and cause cells to undergo G2 phase arrest.

### Cell migration assay

**Fig 11:**
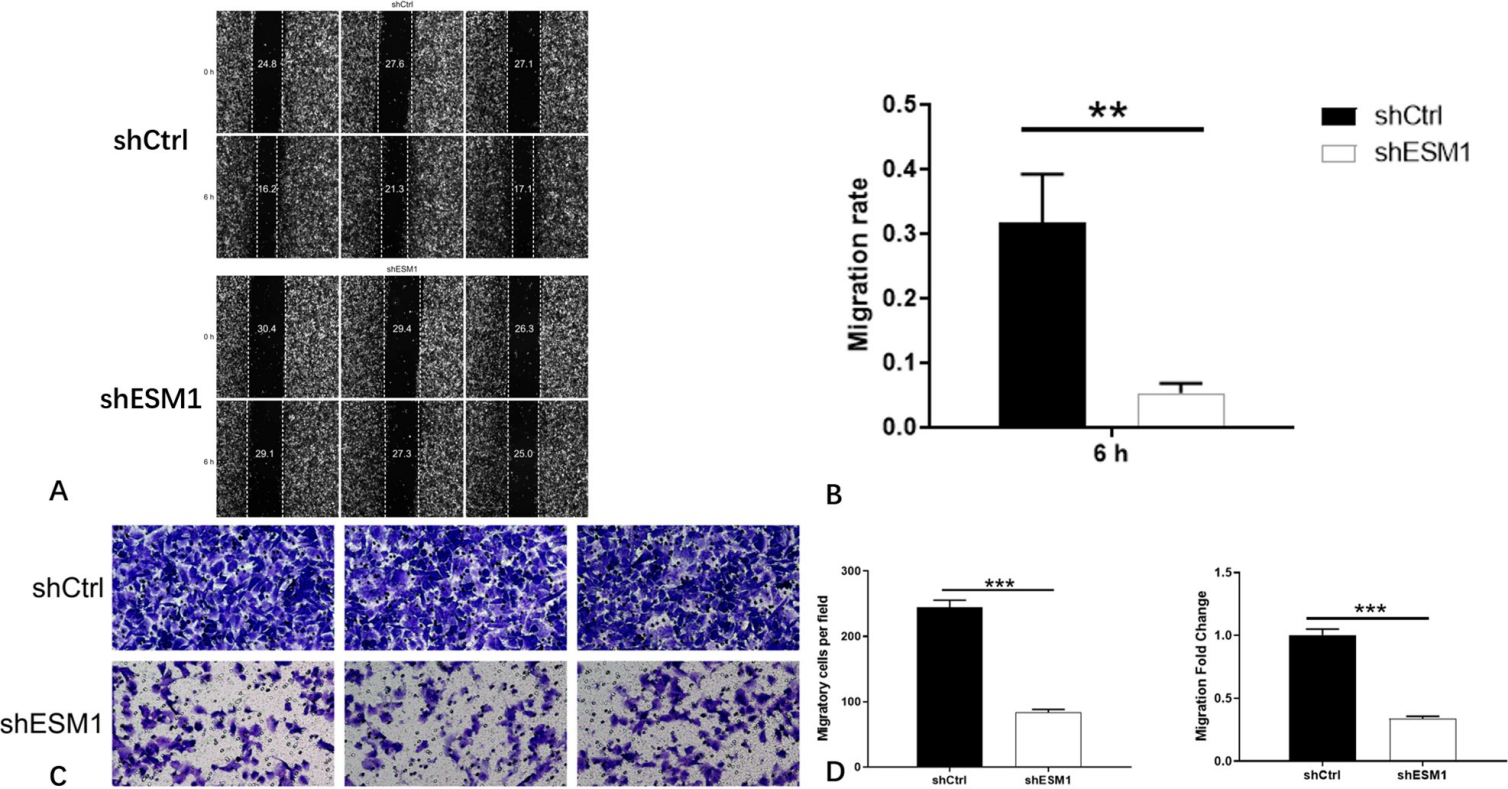
The results of cell migration ability and cell invasion ability of TPC-1 cell lines. A: Cell migration images of shCtrl group and shESM1 group in TPC-1 cells after ESM1 knockdown by scratch assay. B: In TPC-1 cells, the cell migration rate in the shESM1 group decreased by 83% at 6 h compared with the shCtrl group. C: Cell invasion and metastasis images of shCtrl group and shESM1 group in TPC-1 cells after ESM1 knockdown by transwell assay. D: In TPC-1 cells, the invasion and metastasis rate of the shESM1 group was 66% lower than that of the shCtrl group. **P< 0.01, *** P <0.001.

**Fig 12:**
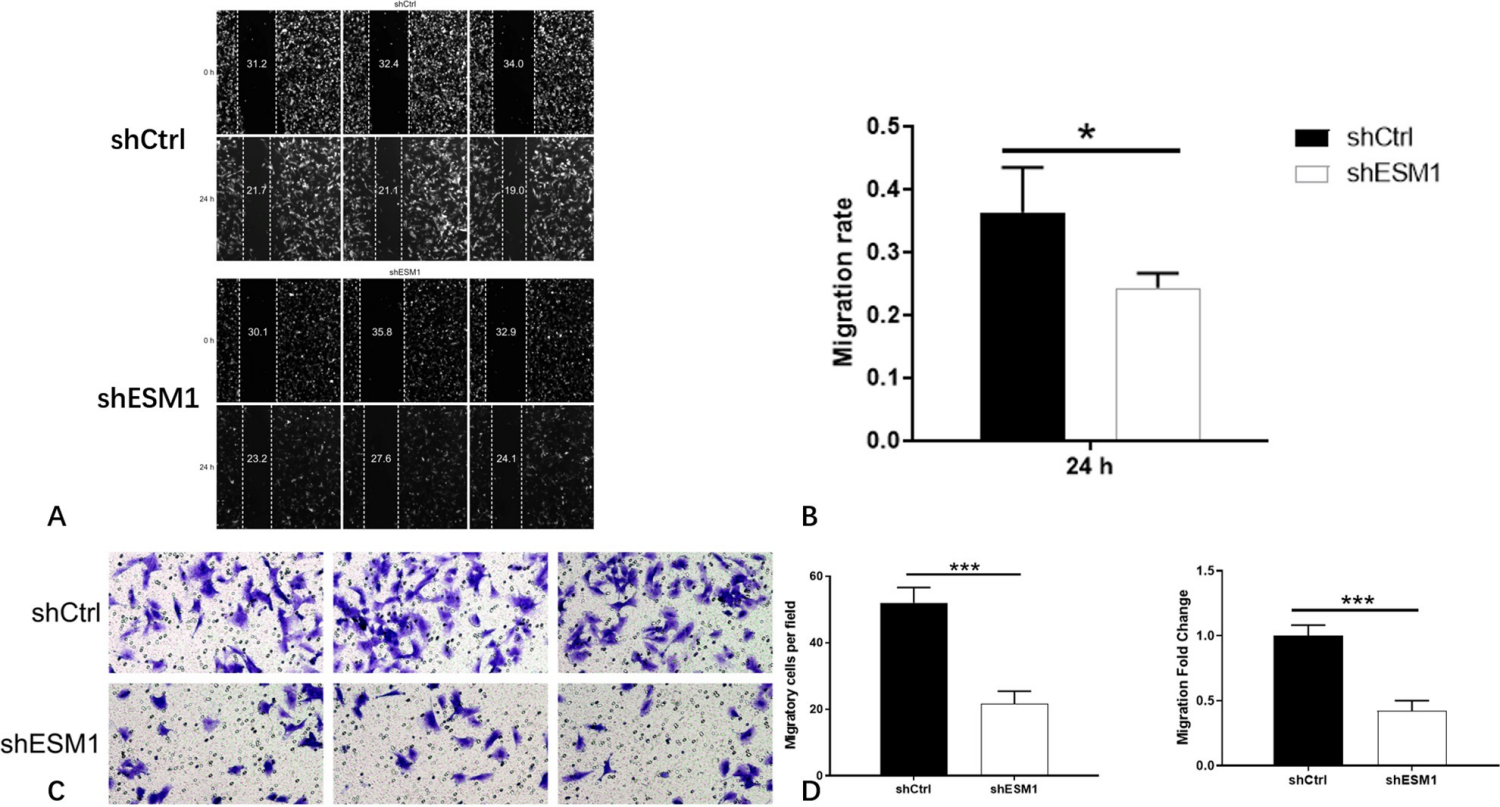
The results of cell migration ability and cell invasion ability of SW579 cell lines. A: Cell migration images of shCtrl group and shESM1 group in SW579 cells after ESM1 knockdown by scratch assay. B: In SW579 cells, the cell migration rate of the shESM1 group decreased by 34% at 24 h compared with the shCtrl group. C: Cell invasion and metastasis images of shCtrl group and shESM1 group in SW579 cells after ESM1 knockdown by transwell assay. D: In SW579 cells, the invasion and metastasis rate of the shESM1 group decreased by 58% compared with the shCtrl group. *P < 0.05, *** P <0.001.

The scratch assay was used to detect the effects of knockdown of ESM1 expression on the migratory ability of thyroid cancer cells. The migratory activity of TPC-1 cells was decreased by 83% at 6 h following their treatment with shESM1 (P<0.01). In SW579, the cell migratory rate of the shESM1 group was decreased by 34% at 24 h (P<0.05). The results indicated that the migratory ability of cells was dramatically inhibited following knockdown of ESM1 expression in TPC-1 and SW579 cells (P<0.05). Concomitantly, the transwell assay was utilized to detect the effect of knockdown of ESM1 expression on the invasive and metastatic ability of thyroid cancer cells. In TPC-1 cells, the invasive and metastatic rate of the shESM1 group was <66% than that of the shCtrl group (P<0.001). In SW579 cells, the invasive and metastatic rate of the shESM1 group was decreased by 58% compared with that of the shCtrl group (P<0.001). The results indicated that following knockdown of ESM1 expression in TPC-1 and SW579 cells, the invasive ability of the cells was significantly inhibited (P<0.001; Figs 11 and 12).

## Discussion

Thyroid cancer, particularly papillary thyroid carcinoma (PTC), is noted for its aggressive metastasis, creating substantial challenges in treatment. While surgical resection, radioactive iodine therapy, and thyroxine replacement therapies have improved patient outcomes, these standard interventions do not always fully eradicate the disease. Recurrence remains a significant issue, often leading to a deterioration in the quality of life for patients [13–15]. This issue underlines the critical necessity for novel therapeutic approaches. Recent molecular studies have shed light on the promise of targeted gene therapy in the management of cancer, offering hope for more effective treatments. One such molecular target that has received increasing attention in oncology is Endothelial cell-specific molecule-1 (ESM1). This molecule has been implicated in various cancers due to its role in mediating inflammation and angiogenesis. Elevated levels of ESM1 have been consistently linked to the altered immune environment of tumor vasculature and increased angiogenesis, essential to tumor growth and metastatic spread [16, 17]. However, the precise relationship between ESM1 and the proliferation and migration of thyroid cancer cells, particularly in PTC, has been insufficiently explored.

Our study aims to fill this research void by revealing that ESM1 levels are significantly higher in thyroid cancer tissues, including PTC, compared to adjacent non-tumorous tissues. By applying RNA interference techniques, specifically shRNA, we have elucidated the functional role of ESM1 in thyroid cancer. Our findings show that ESM1 significantly contributes to the proliferation and migration of tumor cells. Furthermore, the knockdown of ESM1 effectively impedes the growth, migration, and invasion of thyroid cancer cells, indicative of the therapeutic potential observed in other forms of cancer [18–20]. The study notably signifies the first in-depth exploration of ESM1’s role in PTC, emphasizing its overexpression and critical involvement in cell proliferation and migration. Following ESM1 inhibition via shRNA, we observed decreased cellular growth and increased apoptosis rates, supporting ESM1’s value as a therapeutic target [21–24]. The profound impact of ESM1 knockdown on cellular mechanisms not only bolsters its position as a viable prognostic biomarker but also as a potential therapeutic agent in thyroid cancer. Such outcomes reflect the tumor-suppressive effects commonly pursued in cancer research and validate the direction of future investigations. Crucially, PTC is driven by well-characterized oncogenic pathways, such as mutations in the BRAF and RAS genes and rearrangements in RET/PTC, which activate the MAPK/ERK and PI3K/Akt signaling cascades [25]. These pathways are central to the proliferation, survival, and invasion of cancer cells and have been the focus of targeted therapy development [26]. The aberrant expression of ESM1 in PTC could potentially interact with these oncogenic drivers, exacerbating tumor aggressiveness and resistance to standard therapies. By elucidating the role of ESM1 in these pathways, we open new insights into the molecular etiology of PTC and identify potential intervention points for more effective treatments.

Integrating the current standard of care with novel targeted therapies could enhance the management of PTC. For instance, patients who do not respond to radioactive iodine due to dedifferentiation of cancer cells or those who experience significant side effects from standard treatments might benefit from a more personalized approach that includes ESM1-targeted therapies. In light of our findings, ESM1 appears to be a key regulator of thyroid cancer cell biology and could be an invaluable addition to the therapeutic arsenal against PTC, potentially improving patient outcomes and quality of life. Furthermore, the biomarker potential of ESM1 in thyroid cancer diagnosis and prognosis has become increasingly apparent [27]. This diverges from its previously established pro-cancerous role in other malignancies, indicating a more nuanced role for ESM1 in cancer biology. Our study propels ESM1 to the forefront of the discussion on thyroid cancer treatment, suggesting that monitoring ESM1 expression could serve not only as a means of gauging disease progression but also as a guide for customizing patient-specific treatment plans.

The present study elucidates the role of ESM1 in PTC and suggests potential therapeutic implications. However, several limitations warrant attention and suggest directions for future research. Firstly, the sample size utilized in this study was relatively small, which may limit the robustness and the generalizability of our findings. The samples were potentially drawn from a specific demographic or geographical area, which could further limit the applicability of the results across different populations. To corroborate the utility of ESM1 as a therapeutic target, subsequent research should endeavor to include a larger and more diverse cohort of patients, enhancing the external validity of the findings. Secondly, this investigation was conducted in vitro, and while it demonstrated that ESM1 inhibition could attenuate the proliferation and migration of thyroid cancer cells, the clinical relevance of these findings remains to be established. The absence of in vivo validation and the lack of corroborative data from clinical trials mean that the therapeutic potential of targeting ESM1 in patients with PTC cannot be conclusively asserted at this juncture. Future research should incorporate animal models and clinical studies to substantiate the efficacy and safety profile of ESM1-targeted interventions. Thirdly, although the study highlights the involvement of ESM1 in the pathophysiology of PTC and delineates the downstream effects of ESM1 knockdown on tumor cell behavior, the intricate interactions between ESM1 and established oncogenic pathways in PTC. A more detailed mechanistic dissection of how ESM1 intersects with these pathways is necessary to fully understand its role and to pinpoint precise molecular targets for intervention. Further studies are imperative to dissect these molecular relationships and to validate the therapeutic targeting of ESM1 within the broader oncogenic landscape of PTC.

## Conclusions

In conclusion, this investigation highlights ESM1 as an emerging molecular player in the pathology of PTC. By documenting its overexpression and mechanistic involvement in tumor dynamics, the study provides a foundation for the therapeutic application of ESM1 inhibition. The observed effects on cancer cell viability, migration, and morphological integrity following ESM1 knockdown align with the therapeutic goals pursued in other cancer types, marking a milestone in the journey towards targeted treatment strategies for thyroid cancer. As we advance, it is imperative that future research continues to dissect the multifaceted role of ESM1 in cancer biology, advocating for integrated studies across cancer types to solidify its position as a broad-spectrum molecular target in oncology. Next, we will conduct a critical experimental study on the mechanism of the inhibitory and apoptotic effects of ESM1 on thyroid tumors in detail based on the important data and findings of this paper, which will be presented in a separate article.

## Supporting information

S1 Raw images Original, uncropped original blots pictures underlying Fig 3A and 3F from the main text. (PDF)

## References

[1] Shank JB, Are C, Wenos CD. Thyroid Cancer: Global Burden and Trends. Indian J Surg Oncol. 2022 Mar;13(1):40–45. doi: 10.1007/s13193-021-01429-y Epub 2021 Sep 4. ; PMCID: PMC8986939. 35462648 PMC8986939

[2] Prete A, Borges de Souza P, Censi S, Muzza M, Nucci N, Sponziello M. Update on Fundamental Mechanisms of Thyroid Cancer. Front Endocrinol (Lausanne). 2020; 11:102. doi: 10.3389/fendo.2020.00102 32231639 PMC7082927

[3] Bauer AJ. Pediatric Thyroid Cancer: Genetics, Therapeutics and Outcome. Endocrinol Metab Clin North Am. 2020; 49:589–611. doi: 10.1016/j.ecl.2020.08.001 33153669

[4] Bergdorf K, Ferguson DC, Mehrad M, Ely K, Stricker T, Weiss VL. Papillary thyroid carcinoma behavior: clues in the tumor microenvironment. Endocr Relat Cancer. 2019; 26:601–14. doi: 10.1530/ERC-19-0074 30965283 PMC8279427

[5] Zhang H, Shen YW, Zhang LJ, Chen JJ, Bian HT, Gu WJ, et al. Targeting Endothelial Cell-Specific Molecule 1 Protein in Cancer: A Promising Therapeutic Approach. Front Oncol. 2021; 11:687120. doi: 10.3389/fonc.2021.687120 34109132 PMC8181400

[6] Xiao Y, Zhang G, Wang L, Liang M. Exploration and validation of a combined immune and metabolism gene signature for prognosis prediction of colorectal cancer. Front Endocrinol (Lausanne). 2022; 13:1069528. doi: 10.3389/fendo.2022.1069528 36518242 PMC9742469

[7] Kano K, Sakamaki K, Oue N, Kimura Y, Hashimoto I, Hara K, et al. Impact of the ESM-1 Gene Expression on Outcomes in Stage II/III Gastric Cancer Patients Who Received Adjuvant S-1 Chemotherapy. In Vivo. 2020; 34:461–7. doi: 10.21873/invivo.11796 31882514 PMC6984110

[8] Villa E, Critelli R, Lei B, Marzocchi G, Cammà C, Giannelli G, et al. Neoangiogenesis-related genes are hallmarks of fast-growing hepatocellular carcinomas and worst survival. Results from a prospective study. Gut. 2016; 65:861–9. doi: 10.1136/gutjnl-2014-308483 25666192

[9] van Velsen E, Visser WE, Stegenga MT, Mäder U, Reiners C, van Kemenade FJ, et al. Finding the Optimal Age Cutoff for the UICC/AJCC TNM Staging System in Patients with Papillary or Follicular Thyroid Cancer. Thyroid. 2021; 31:1041–9. doi: 10.1089/thy.2020.0615 33487121

[10] Firek AA, Perez MC, Gonda A, Lei L, Munir I, Simental AA, et al. Pathologic significance of a novel oncoprotein in thyroid cancer progression. Head Neck. 2017; 39:2459–69. doi: 10.1002/hed.24913 29024261

[11] Zhao H, Guo Y, Sun Y, Zhang N, Wang X. miR-181a/b-5p ameliorates inflammatory response in monocrotaline-induced pulmonary arterial hypertension by targeting endocan. J Cell Physiol. 2020; 235:4422–33. doi: 10.1002/jcp.29318 31637717

[12] Zhou R, Wang G, Li Q, Meng F, Liu C, Gan R, et al. A signalling pathway for transcriptional regulation of sleep amount in mice. Nature. 2022; 612:519–27. doi: 10.1038/s41586-022-05510-6 36477534

[13] Roman BR, Randolph GW, Kamani D. Conventional Thyroidectomy in the Treatment of Primary Thyroid Cancer. Endocrinol Metab Clin North Am. 2019; 48:125–41. doi: 10.1016/j.ecl.2018.11.003 30717897

[14] Pace-Asciak P, Russell JO, Tufano RP. The Treatment of Thyroid Cancer With Radiofrequency Ablation. Tech Vasc Interv Radiol. 2022; 25:100825. doi: 10.1016/j.tvir.2022.100825 35551804

[15] Nabhan F, Dedhia PH, Ringel MD. Thyroid cancer, recent advances in diagnosis and therapy. Int J Cancer. 2021; 149:984–92. doi: 10.1002/ijc.33690 34013533

[16] Zhang H, Shen YW, Zhang LJ, Chen JJ, Bian HT, Gu WJ, et al. Targeting Endothelial Cell-Specific Molecule 1 Protein in Cancer: A Promising Therapeutic Approach. Front Oncol. 2021; 11:687120. doi: 10.3389/fonc.2021.687120 34109132 PMC8181400

[17] Liu X, Wang F, Du W, Yang X. ESM-1 Mediates Cell Progression in Clear Cell Renal Cell Carcinoma by Affecting Wnt/β-Catenin Signalling Pathway. Arch Esp Urol. 2023;76(4):290–297. doi: 10.56434/j.arch.esp.urol.20237604.33 37455528

[18] Cai L, Leng ZG, Guo YH, Lin SJ, Wu ZR, Su ZP, et al. Dopamine agonist resistance-related endocan promotes angiogenesis and cells viability of prolactinomas. Endocrine. 2016; 52:641–51. doi: 10.1007/s12020-015-0824-2 26662185

[19] Kang N, Liang X, Fan B, Zhao C, Shen B, Ji X, et al. Endothelial-Specific Molecule 1 Inhibition Lessens Productive Angiogenesis and Tumor Metastasis to Overcome Bevacizumab Resistance. Cancers (Basel). 2022; 14:5681. doi: 10.3390/cancers14225681 36428773 PMC9688485

[20] Li YK, Zeng T, Guan Y, Liu J, Liao NC, Wang MJ, et al. Validation of ESM1 Related to Ovarian Cancer and the Biological Function and Prognostic Significance. Int J Biol Sci. 2023; 19:258–80. doi: 10.7150/ijbs.66839 36594088 PMC9760436

[21] Li C, Geng H, Ji L, Ma X, Yin Q, Xiong H. ESM-1: A Novel Tumor Biomaker and its Research Advances. Anticancer Agents Med Chem. 2019; 19:1687–94. doi: 10.2174/1871520619666190705151542 31284875

[22] Gu X, Zhang J, Shi Y, Shen H, Li Y, Chen Y, et al. ESM1/HIF‑1α pathway modulates chronic intermittent hypoxia‑induced non‑small‑cell lung cancer proliferation, stemness and epithelial‑mesenchymal transition. Oncol Rep. 2021; 45:1226–34. doi: 10.3892/or.2020.7913 33650648

[23] Huang YG, Wang Y, Zhu RJ, Tang K, Tang XB, Su XM. EMS1/DLL4-Notch Signaling Axis Augments Cell Cycle-Mediated Tumorigenesis and Progress in Human Adrenocortical Carcinoma. Front Oncol. 2021; 11:771579. doi: 10.3389/fonc.2021.771579 34858850 PMC8631517

[24] Lu J, Liu Q, Zhu L, Liu Y, Zhu X, Peng S, et al. Endothelial cell-specific molecule 1 drives cervical cancer progression. Cell Death Dis. 2022; 13:1043. doi: 10.1038/s41419-022-05501-5 36522312 PMC9755307

[25] Dong X, Akuetteh PDP, Song J, Ni C, Jin C, Li H, et al. Major Vault Protein (MVP) Associated With BRAFV600E Mutation Is an Immune Microenvironment-Related Biomarker Promoting the Progression of Papillary Thyroid Cancer via MAPK/ERK and PI3K/AKT Pathways. Front Cell Dev Biol. 2022; 31;9:688370. doi: 10.3389/fcell.2021.688370 35433709 PMC9009514

[26] Zhou Q, Chen J, Feng J, Xu Y, Zheng W, Wang J. SOSTDC1 inhibits follicular thyroid cancer cell proliferation, migration, and EMT via suppressing PI3K/Akt and MAPK/Erk signaling pathways. Mol Cell Biochem. 2017;435(1–2):87–95. doi: 10.1007/s11010-017-3059-0 28551845

[27] Lai CY, Chen CM, Hsu WH, Hsieh YH, Liu CJ. Overexpression of Endothelial Cell-Specific Molecule 1 Correlates with Gleason Score and Expression of Androgen Receptor in Prostate Carcinoma. Int J Med Sci. 2017; 14:1263–7. doi: 10.7150/ijms.21023 . 29104483 PMC5666560

