# Supplementary material for: Functional analysis of ESM1 by shRNA-mediated knockdown of its expression in papillary thyroid cancer cells
Source: PLoS One. 2024 Apr 16;19(4):e0298631. doi: 10.1371/journal.pone.0298631 (PMC11020426; doi:10.1371/journal.pone.0298631)
Supplement: S1 Raw images — (PDF) [file pone.0298631.s001.pdf]

Supplementary explanations for Figure 3C and 3F

Supplementary explanations for Figure 3C:

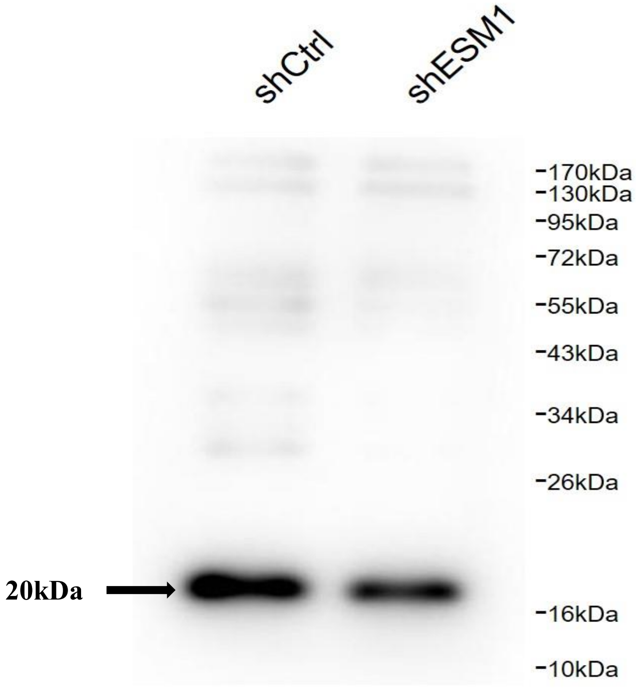

TPC-1-ESM1

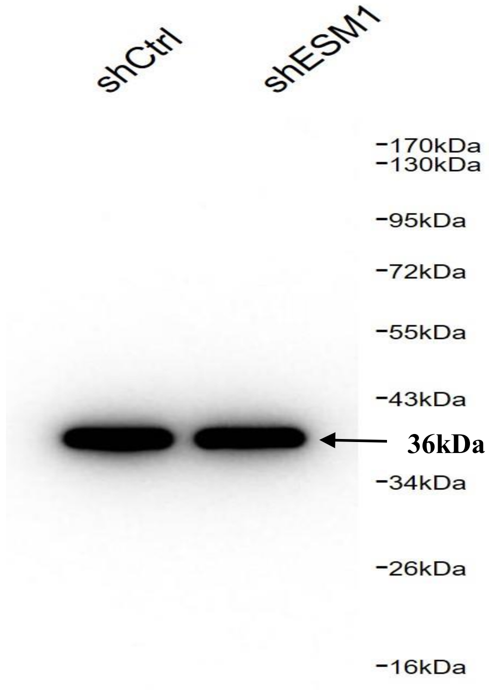

TPC-1-GAPDH

Supplementary explanations for Figure 3F:

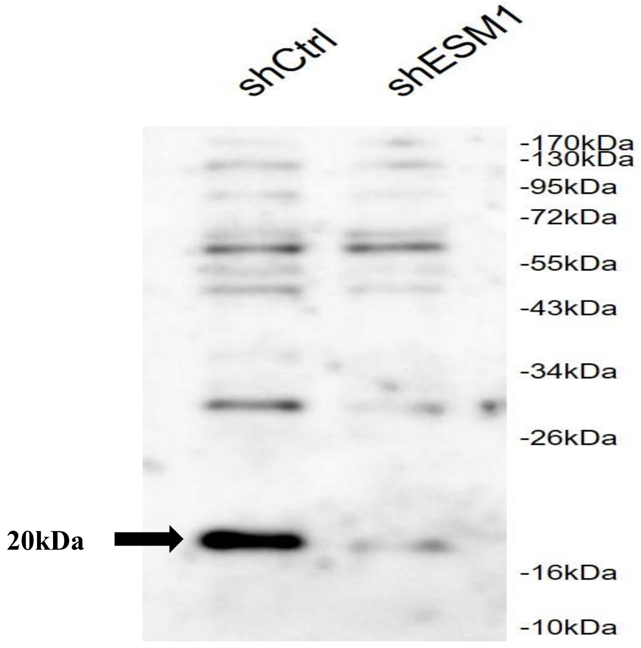

SW579-ESM1

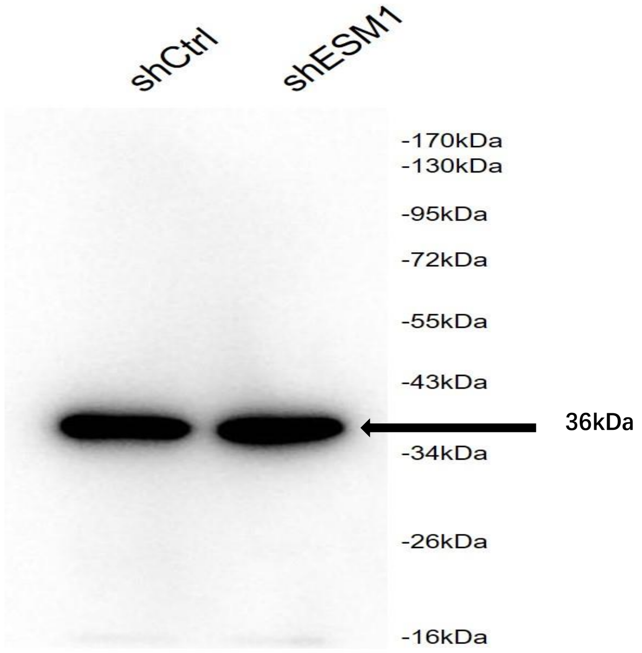

SW579-GAPDH
